# Comparative Analysis of Age- and Gender-Associated Microbiome in Lung Adenocarcinoma and Lung Squamous Cell Carcinoma

**DOI:** 10.3390/cancers12061447

**Published:** 2020-06-02

**Authors:** Lindsay M. Wong, Neil Shende, Wei Tse Li, Grant Castaneda, Lauren Apostol, Eric Y. Chang, Weg M. Ongkeko

**Affiliations:** 1 Department of Otolaryngology-Head and Neck Surgery, University of California, La Jolla, San Diego, CA 92093, USA; lmw010@ucsd.edu (L.M.W.); nshende@ucsd.edu (N.S.); wtl008@ucsd.edu (W.T.L.); gecastan@ucsd.edu (G.C.); laaposto@ucsd.edu (L.A.); 2 Research Service, VA San Diego Healthcare System, San Diego, CA 92161, USA; 3 Department of Radiology, University of California, San Diego, CA 92093, USA; eric.chang2@va.gov; 4 Radiology Service, VA San Diego Healthcare System, San Diego, CA 92161, USA

**Keywords:** lung adenocarcinoma, lung squamous cell carcinoma, microbiome, age, gender, TCGA

## Abstract

The intra-tumor microbiota has been increasingly implicated in cancer pathogenesis. In this study, we aimed to examine the microbiome in lung adenocarcinoma (LUAD) and lung squamous cell carcinoma (LUSC) and determine its compositional differences with relation to age and gender. After grouping 497 LUAD and 433 LUSC patients by age and gender and removing potential contaminants, we identified differentially abundant microbes in each patient cohort vs. adjacent normal samples. We then correlated dysregulated microbes with patient survival rates, immune infiltration, immune and cancer pathways, and genomic alterations. We found that most age and gender cohorts in both LUAD and LUSC contained unique, significantly dysregulated microbes. For example, LUAD-associated *Escherichia coli* str. K-12 substr. W3110 was dysregulated in older female and male patients and correlated with both patient survival and genomic alterations. For LUSC, the most prominent bacterial species that we identified was *Pseudomonas putida* str. KT2440, which was uniquely associated with young LUSC male patients and immune infiltration. In conclusion, we found differentially abundant microbes implicated with age and gender that are also associated with genomic alterations and immune dysregulations. Further investigation should be conducted to determine the relationship between gender and age-associated microbes and the pathogenesis of lung cancer.

## 1. Introduction

The leading cause of cancer-related deaths is lung cancer [1], which can be divided into small-cell lung cancers and non-small-cell lung cancers. Accounting for 85% of lung cancers [2], non-small-cell lung cancers can be further subdivided into lung adenocarcinoma (LUAD), lung squamous cell carcinoma (LUSC), and large-cell lung cancer [3]. LUAD accounts for about 40% of lung cancer cases [4], whereas LUSC accounts for around 25% of non-small-cell lung cancer occurrences [3]. Overall, the 5-year survival rate of lung cancer is only 17% [4]. The broad impact and low survival rates of lung cancer highlight the critical importance of studying the etiologies and risk factors for lung cancer. 

The human lungs have a diverse microbiota that is composed of many types of organisms. One study found over nine genera of bacteria in the lungs [5,6]. It has been found that the microbiome plays a significant role in disease and immunity in the lungs [6]. Furthermore, studies have supported an association between the microbiome and cancer, including lung cancer [7]. Factors including age have been implicated in the microbiome where it has been discovered that community diversity lowers in older age groups when specifically studying the lung microbiome [8,9]. Gender also plays a significant role in regards to the microbiome, as the microbiota associated with each gender is unique because of the hormones specific to each gender [10]. Although there are many studies conducted on LUAD and LUSC, relatively few focus on the association of these cancers with their respective microbiome [11]. 

Clinical variables, including age and gender, play significant roles in the development of cancers. Approximately 90% of lung cancer cases occur in individuals over the age of 55, whereas only around 10% of lung cancer cases occur in individuals under the age of 55 [12]. Younger lung cancer patients also tend to have higher survival rates. This difference in cancer incidence and patient survival rates indicates that age could determine the microbiome, which, in turn, regulates the pathogenesis and progression of LUAD and LUSC. Gender is also an important factor to consider in cancer pathogenesis. Men tend to have higher mortality rates and a higher incidence of lung cancer than women. This could be explained partially by the fact that men tend to smoke more tobacco. Curiously, incidence of lung cancer is higher in non-smoking women than in non-smoking men [13]. Younger lung cancer patients are also more likely to be female [12]. Thus, it is imperative to investigate and characterize the association between the lung microbiome and lung cancers such as LUAD and LUSC in the context of both age and gender. In this study, we investigated the lung microbiome in LUAD and LUSC patients by dividing patients into eight cohorts based on age and gender and comparing the presence of similar or unique bacteria across cohorts. From these comparisons, we correlated the abundance of these microbes to patient survival, immune cell populations, immune signatures, immune and cancer pathways, and genomic alterations.

## 2. Results

### 2.1. Aligning Microbial Reads and Initial Comparison of Cohorts

**Figure 1:**
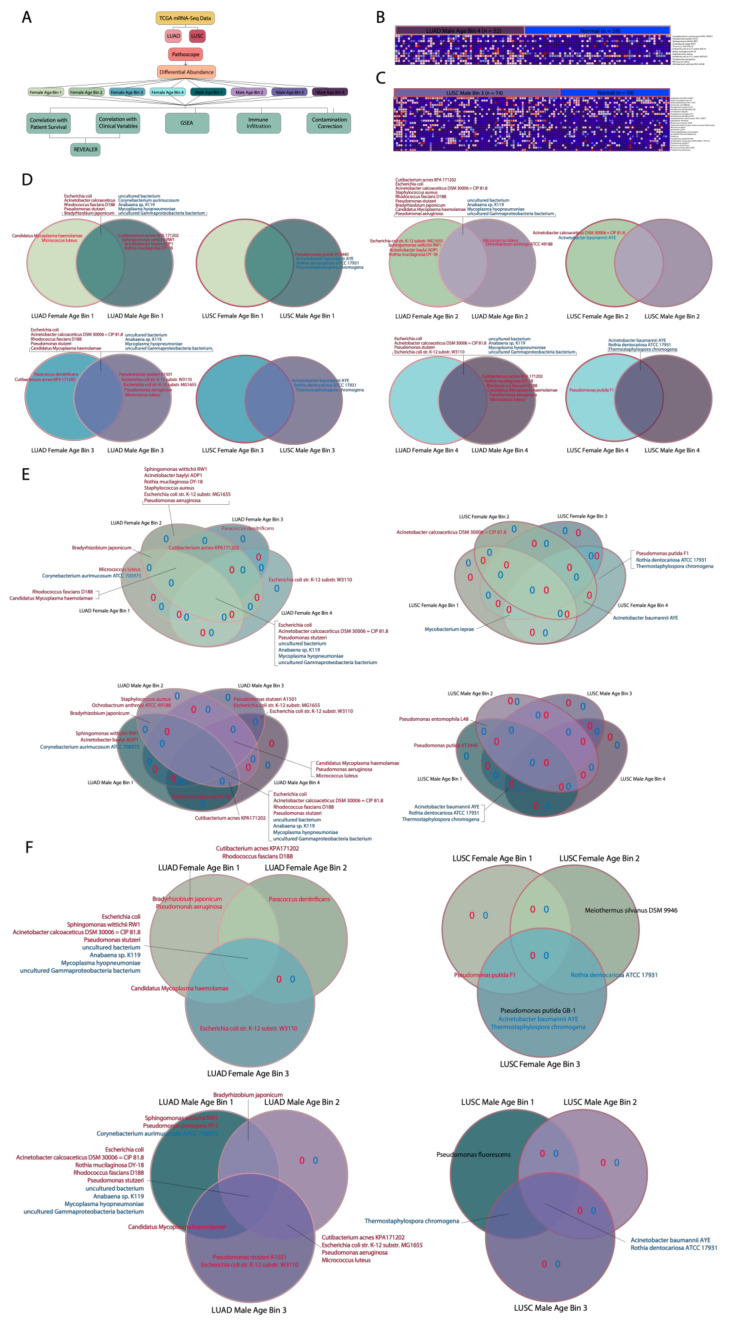
Summary of differential abundance and patient cohort comparison results. (**A**) Schematic of study workflow. (**B**) Heat map comparing the lung adenocarcinoma (LUAD) Male Age Bin 4 cohort to adjacent normal samples. (**C**) Heat map comparing the lung squamous cell carcinoma (LUSC) Male Age Bin 3 cohort to corresponding adjacent normal samples. (**D**) Two-way Venn Diagram comparing the microbes in female and male cohorts within one cancer. Microbes written with red text represent upregulated microbes, whereas microbes written with blue text represent downregulated microbes when comparing cancer cohorts to normal samples. (**E**) Four-way Venn Diagram comparing the microbes associated with each age bin for each gender. Only microbes differentially abundant in LUSC or LUAD samples vs. normal samples are included (Kruskal–Wallis test, *p* < 0.05). Microbes written with red text represent upregulated microbes, whereas microbes written with blue text represent downregulated microbes when comparing cancer cohorts to normal samples. (**F**) Three-way Venn Diagram comparing the microbes associated with each age bin for each gender. Only microbes differentially abundant in LUSC or LUAD samples vs. normal samples are included (Kruskal–Wallis test, *p* < 0.05). Microbes written with red text or blue text represent upregulated and downregulated microbes, respectively, when comparing cancer cohorts to normal samples. Red or blue text also indicates that these microbes are also found in the four-way Venn diagram, while black text indicates that these microbes indicate that these microbes could not be located in the four-way Venn Diagram.

Pathoscope 2.0 was performed to align and quantify the reads of microbial species using TCGA mRNA sequencing data from lung adenocarcinoma (LUAD) and lung squamous cell carcinoma (LUSC) samples (Figure 1A) [14]. TCGA samples were collected during surgery, and no prior treatments (chemotherapy, radiotherapy, immunotherapy, etc.) were given prior to the surgery. After separating the patients into eight cohorts based on age and gender for each cancer, we compared the data of cancer cohorts within one cancer directly to each other and to adjacent normal samples (Kruskal–Wallis test). Compared to cancer vs. normal comparisons, comparisons between two different cohorts of cancer patients did not have many microbes with significant correlations (Table S1). Thus, we proceeded with further analyses using patients’ microbial abundance data from the cancer cohorts (LUAD Female Age Bin 1, 2, 3, 4, LUAD Male Age Bin 1, 2, 3, 4, LUSC Female Age Bin 1, 2, 3, 4, LUSC Male Age Bin 1, 2, 3, 4) vs. corresponding normal (LUAD normal samples or LUSC normal samples) comparisons (Figure 1B,C). Age bins were created using the first, second and third quartile of male and female ages within an individual cancer. Ages between the minimum age to first quartile, the first quartile to the second quartile, the second quartile to the third quartile and third quartile to the maximum age corresponded to the first, second, third and fourth age bin, respectively. To validate whether our results are affected by this age grouping, we also performed analyses with three instead of four age groups.

### 2.2. Determination of Age- and Gender-Associated Microbes

To reveal the dysregulated microbes associated with age and gender, we compared significantly dysregulated microbes between genders within the same age bins (i.e., Male Age Bin 1 vs. Female Age Bin 1) and between age bins within the same gender (i.e., Female Age Bin 1 vs. Female Age Bin 2 vs. Female Age Bin 3 vs. Female Age Bin 3) for each cancer. 

We found that in the LUAD gender comparisons, all LUAD female and male age bins contained uniquely implicated microbes except for LUAD Female Age Bin 4 (73–88 years), which had none (Figure 1D). In the LUSC gender comparisons, two LUSC female age bins contained uniquely implicated microbes, which were LUSC Female Age Bin 2 (62–67 years) and 4 (73–85 years). Interestingly, the opposite holds true for LUSC male age bins where only LUSC Male Age Bin 1 (45–61 years) and 3 (68–72 years) contained uniquely implicated microbes. The only LUSC gender comparison that had microbes in common between their female and male age bins was LUSC Female Age Bin 4 and Male Age Bin 4, which both shared *Acinetobacter baumannii* str. AYE, *Rothia dentocariosa* str. ATCC 17931, and *Thermostaphylospora chromogena*. 

For the LUAD age bin comparisons between female cohorts, all cohorts had at least one uniquely associated microbe, with LUAD Female Age Bin 2 (33–58 years) containing the most uniquely associated microbes with high abundance (Figure 1E). There were not many female age bins that shared microbes with one or two other female age bins. For the LUAD age bin comparisons between male cohorts, all contained uniquely associated microbes except for LUAD Male Age Bin 4 (73–88 years). For LUSC female age bin comparisons, LUSC Female Age Bin 2 (*A. calcoaceticus* str. DSM 20,006 = CIP 81.8) and LUSC Female Age Bin 4 (*Pseudomonas putida* str. F1, *R. dentocariosa* str. ATCC 17931, *T. chromogena*) contained uniquely associated microbes. On the other hand, only LUSC Male Age Bin 1 contained uniquely implicated microbes (*P. putida str. KT2440*) while more microbes were shared between male cohorts, including LUSC Male Age Bin 1 and 2 (*Pseudomonas entomophila* str. L48) and LUSC Male Age Bin 1, 3, and 4 (*A. baumannii* str. AYE, *R. dentocariosa* str. ATCC 17931 and *T. chromogena*).

We obtained very similar results when patients are grouped into three age groups instead of four age groups in that microbes associated with older patients for 4-age-bin comparisons are also associated with older patients for 3-age-bin comparisons, and the same holds true for younger patients (Figure 1F). This suggests that the age-related correlations are not an artifact of patient binning. We also sought to identify potential confounding variables by correlating microbes we found dysregulated in the different age and gender groups to other clinical variables using logistic regression (Table S2). We found that most microbes exhibit no correlations with clinical variables, including ethnicity, race, smoking history, and tumor stage. If a confounding variable exists, we would expect such a variable to consistently correlate with the abundance of microbes we identified. 

Collectively, our results suggest that many microbes are dysregulated only in certain age bins or genders, which is evidence for the possibility that age and gender may be factors in determining a cancer patient’s lung microbiome. However, age seems to be a greater factor in determining the microbiome composition in LUAD, compared to LUSC. 

### 2.3. Identification of Potential Microbial Contaminants

**Figure 2:**
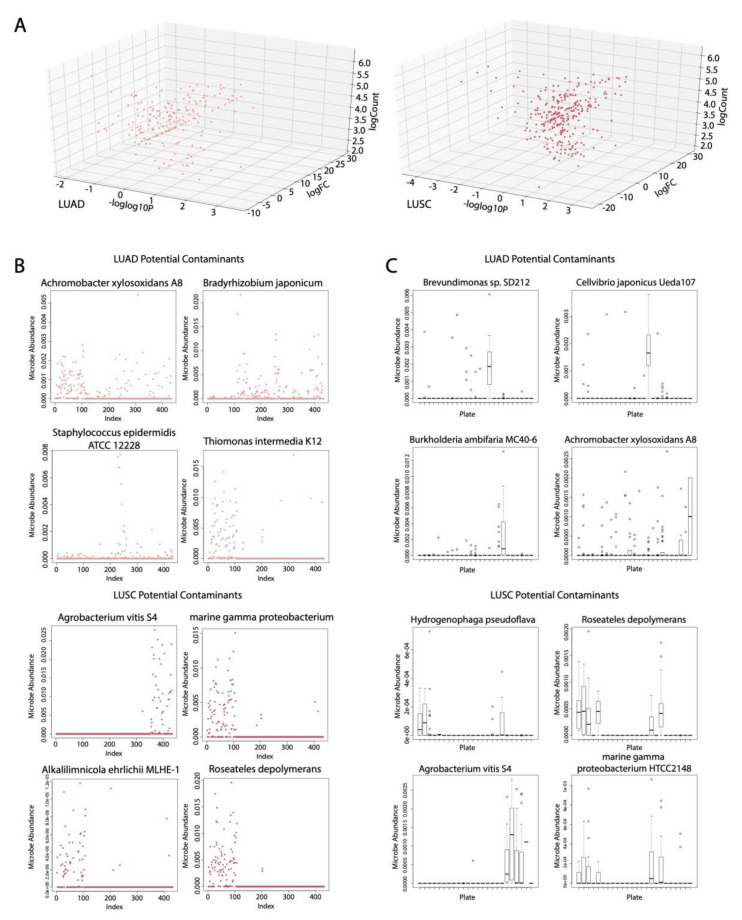
Contamination correction results. (**A**) Differentially abundant microbes from comparing cancer samples vs. normal samples in LUAD and LUSC (Kruskal–Wallis test, *p* < 0.05). (**B**) Dot plot visualizing potential microbial contaminants according to date sequenced using an algorithm that relies on divisive clustering of abundance for LUAD and LUSC. (**C**) Boxplots depicting potential microbial contaminants according to the plate ID for LUAD and LUSC (*p* < 0.05).

We corrected for microbial contamination using the abundance values of microbes associated with plates and sequencing dates to obtain a list of potential contaminants for LUAD and LUSC. Following comparison of cancer cohorts to normal using the Kruskal–Wallis test for both LUAD and LUSC, we found that most significant microbes were not potential contaminants because of their high fold change values (Figure 2A). We reached this conclusion by assuming that cancer and normal samples are handled similarly, so if a microbe is an environmental contaminant, its fold change would be similar between cancer and normal samples.

From visualizing the same microbes in a heat map where the patient samples are ordered by the date sequenced, we found that, in LUSC, there were many repetitive patterns in the heat map that we attributed to potential microbial contaminants (Figure S1). We removed all microbes that we identified as potential contaminants from all analyses. For the microbes identified as contaminants, we visualized the abundance of selected microbes according to the date sequenced (Figure 2B). Because the abundance of potential microbial contaminants is much more enriched on some sequencing dates than on others, this data suggest that, on these particular dates, the microbes have contaminated the sequenced samples. The potential microbial contaminants with the most pronounced spike in abundance enrichment for a certain sequencing date or several sequencing dates include *Achromobacter xylosoxidans* str. A8, *Bradyrhizobium japonicum*, *Staphylococcus epidermidis* str. ATCC 12228, and *Thiomonas intermedia* str. K12 for LUAD and *Agrobacterium vitis* str. S4, *marine gamma proteobacterium*, *Alkalilimnicola ehrlichii* str. MLHE-1 and *Roseateles deploymerans* for LUSC. We also visualized selected potential contaminants according to the microbial abundance associated with sample plates, as documented by TCGA, using boxplots (Figure 2C). Microbes that had abundance values conspicuously higher in certain plates than the rest of the plates were considered to be likely contaminants. We selected *Brevundimonas* sp. SD212, *Cellvibrio japonicus* str. Ueda 107, *Burkholderia ambifaria* str. MC40-6 and *A. xylosoxidans* A8 for LUAD and *Hydrogenophaga pseudoflava*, *R. depolymerans*, *A. vitis* str. S4 and *marine gamma proteobacterium* str. HTCC2148 for display because of their more pronounced boxplots. 

### 2.4. Contributions of Age and Gender to the Landscape of Microbe Abundances

**Figure 3:**
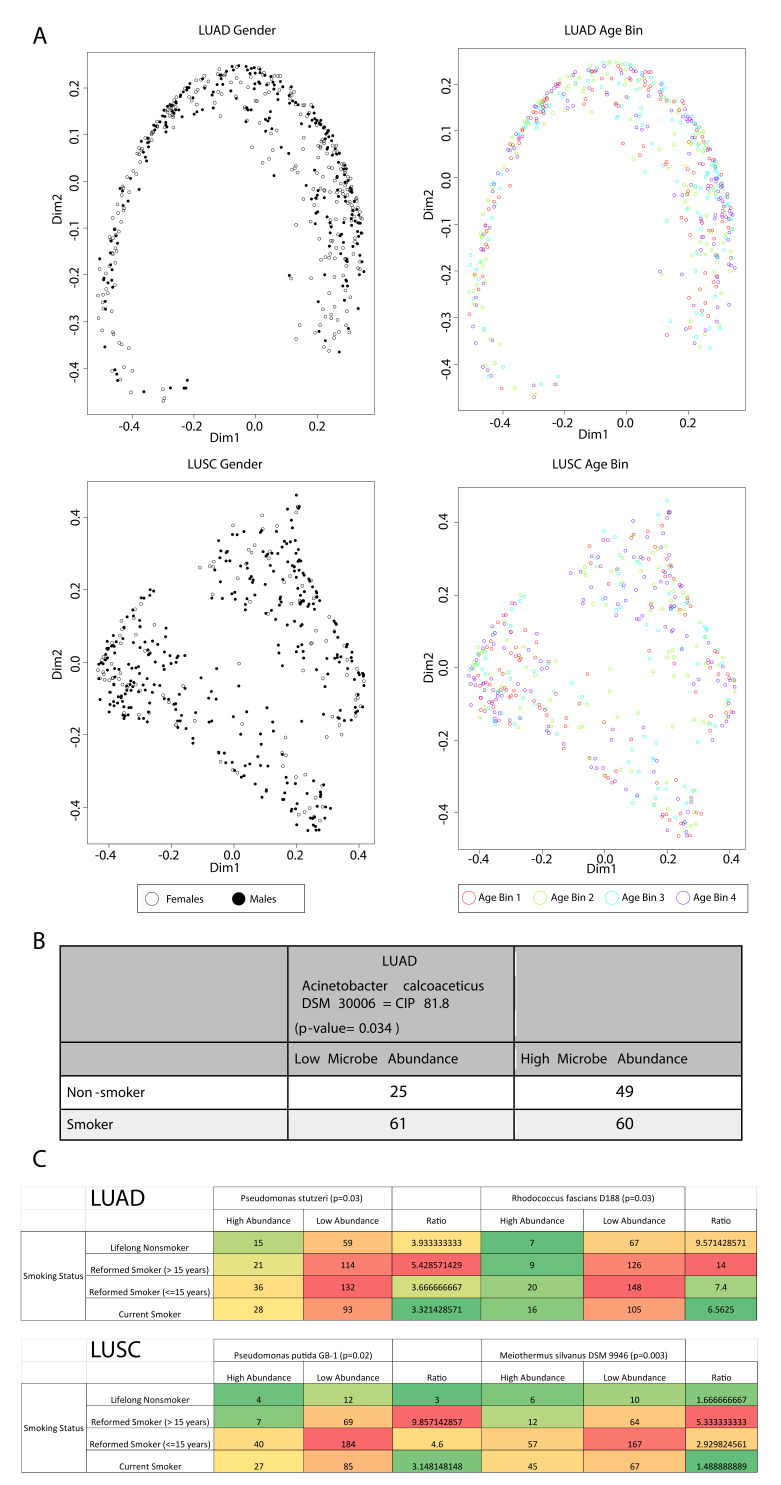
Principle coordinates analysis (PCoA) plot and smoking correlations. (**A**) PCoA plot demonstrating difference in microbial abundance between age and gender in LUAD and LUSC. (**B**) Smoking correlation with the LUAD-associated microbe *Acinetobacter calcoaceticus* DSM 300,006 = CIP 81.8 abundance (χ^2^ test, *p* < 0.05). (**C**) Smoking correlation with the abundance of microbes with reformed smokers (logistic regression, *p*-value < 0.05).

After the removal of potential contaminants, we computed the Bray–Curtis dissimilarity matrix for all cancer samples and then visualized the dissimilarity between samples using a principle coordinates analysis (PCoA) plot (Figure 3A). A PCoA plot reduces the dimension of variables (microbes), so similarities and differences in the entire microbiome landscape can be visualized in a 2D plot. The closer a point is to another point, the more similar in microbe abundances the two points are. We discovered that there is a parabolic pattern on the PCoA plot for LUAD, which is known as the arch or horseshoe effect (Figure 3A). While this effect could be an artifact of dimensionality reduction, it has also been suggested that this pattern is indicative of a strong linear correlation of microbe abundance with a variable [15]. While this variable may be an actual clinical variable, it is also highly likely that it is a latent variable that does not have physical interpretations. We found that neither gender nor age is the variable defining this parabolic pattern. However, gender has a larger effect on the microbiome landscape in LUAD than age does, as evidenced by the observation that, in the positive region of Dimension 1, female samples are more likely to be on the inner side of the parabola, and male samples are more likely to be on the outer side of the parabola (Figure 3A). Neither age nor gender has a visible effect on the microbe abundance landscape of LUSC (Figure 3B). 

### 2.5. Correlation of Age or Gender-Associated Microbes’ Abundances to Smoking History

We found that only *Acinetobacter calcoaceticus* in LUAD exhibited correlations with smokers vs. nonsmokers (χ^2^ test, *p* < 0.05) (Figure 3B). Two microbes in LUAD, *Pseudomonas stutzeri* and *Rhodococcus fascians* str. D188 and two microbes in LUSC, *Pseudomonas putida* str. GB-1 and *Meiothermus silvanus* str. DSM 9946, exhibited correlations with reformed smokers (Figure 3C) (logistic regression, *p* < 0.05). We presented contingency tables for all correlations, which should be interpreted through ratios of numbers in the low abundance column to numbers in the high abundance column. Absolute numbers may be misleading because some microbes have unequal numbers of samples in the LOW and HIGH groups. A low abundance of *A. calcoaceticus* was correlated with smoking in LUAD (Figure 3B). A low abundance of *P. stutzeri and R. fascians* str. D188 in LUAD are correlated with nonsmokers and smokers reformed for over 15 years, suggesting that higher abundance is correlated with smoking for these microbes (Figure 3C). A low abundance of *P*. *putida* str. GB-1 and *M. silvanus* str. DSM 9946 in LUSC are strongly correlated with smokers reformed for over 15 years but not with nonsmokers, but this could be a result of small patient numbers for nonsmokers (n = 16). 

### 2.6. Correlation of Significant Microbes to Patient Survival

**Figure 4:**
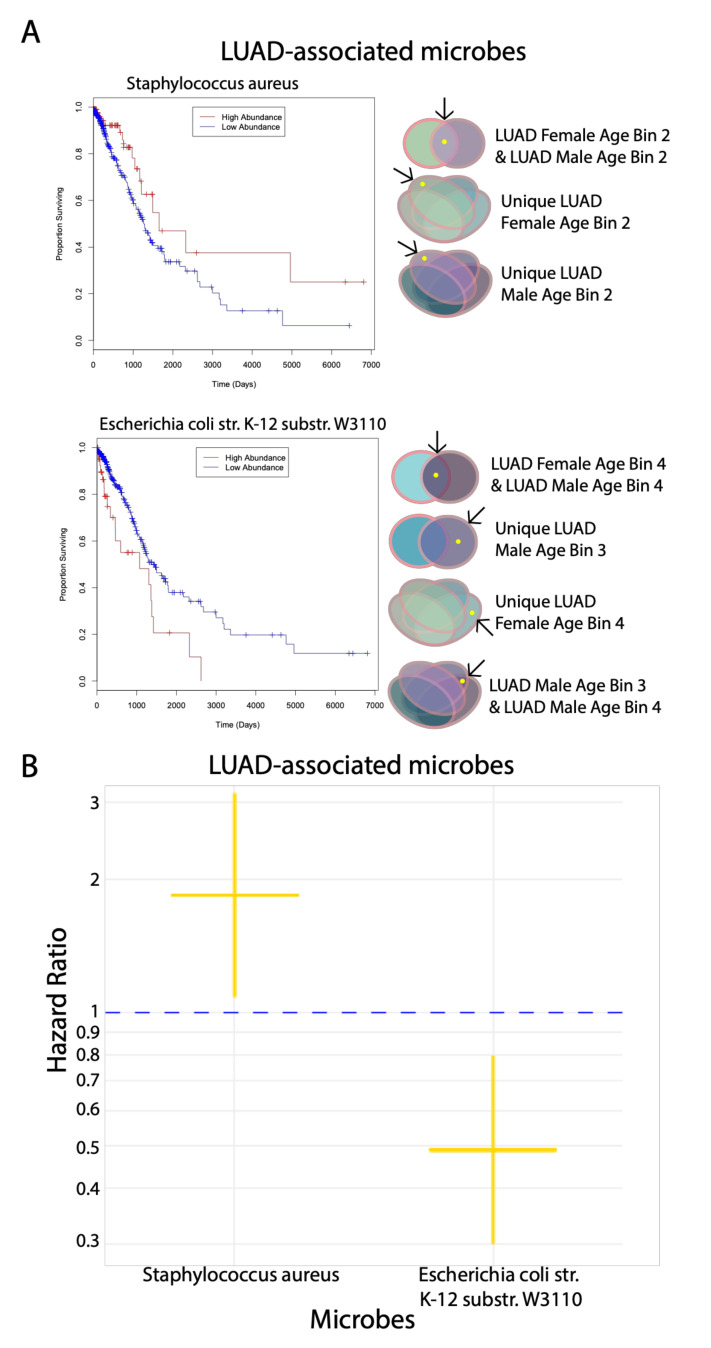
Correlation of differentially abundant microbes in LUAD to patient survival. (**A**) Kaplan–Meier plots of differentially abundant microbes in LUAD. The comparisons and cohorts that these microbes are associated with are shown to the side. (**B**) Hazard ratio plot of differentially abundant microbes in LUAD. Above the cut-off of 1 demonstrates that lower expression of the microbe correlates with lower patient survival, while below the cut-off of 1 demonstrates that the lower expression of the microbe correlates to greater patient survival.

For all significant microbes that we found following the gender and age bin comparisons of gender and age bins, respectively, for both cancers, we correlated the abundance of these microbes to patient survival using the Cox proportional hazards regression model (*p* < 0.05). Out of the significant differentially abundant microbes for LUAD and LUSC, only two microbes for LUAD (*Staphylococcus aureus* and *E. coli* str. K-12 substr. W3110) were found to correlate significantly with patient survival, which we visualized using Kaplan–Meier plots (Figure 4A). Higher abundance of *S. aureus* and lower abundance of *E. coli* str. K-12 substr. W3110 correlate with increased patient survival, whereas the opposite abundance levels correlate with decreased patient survival. This observation suggests that *S. aureus* and *E. coli* str. K-12 substr. W3110 play a tumor-suppressive and oncogenic role, respectively. *S. aureus* is found to be in common between both LUAD Female and Male Age Bin 2 in the gender comparisons and is associated uniquely with LUAD Female Age Bin 2 and LUAD Male Age Bin 2 in the age bin comparisons. *E. coli* str. K-12 substr. W3110 was discovered to be in common with LUAD Female and Male Age Bin 4 and uniquely associated with LUAD Male Age Bin 3 in the gender comparisons (Figure 1D). In the age bin comparisons, *E. coli* str. K-12 substr. W3110 was also found to be uniquely associated with LUAD Female Age Bin 4 and in common between LUAD Male Age Bin 3 and 4 (Figure 1E).

The hazard ratios for the two microbes signify that patients with high abundance of *S. aureus* are twice as likely to survive, whereas patients with high abundance of *E. coli* str. K-12 substr. W3110 are more likely to die by around two-fold (Figure 4B). 

### 2.7. Association of Immune Cell Populations to Significant Microbes

**Figure 5:**
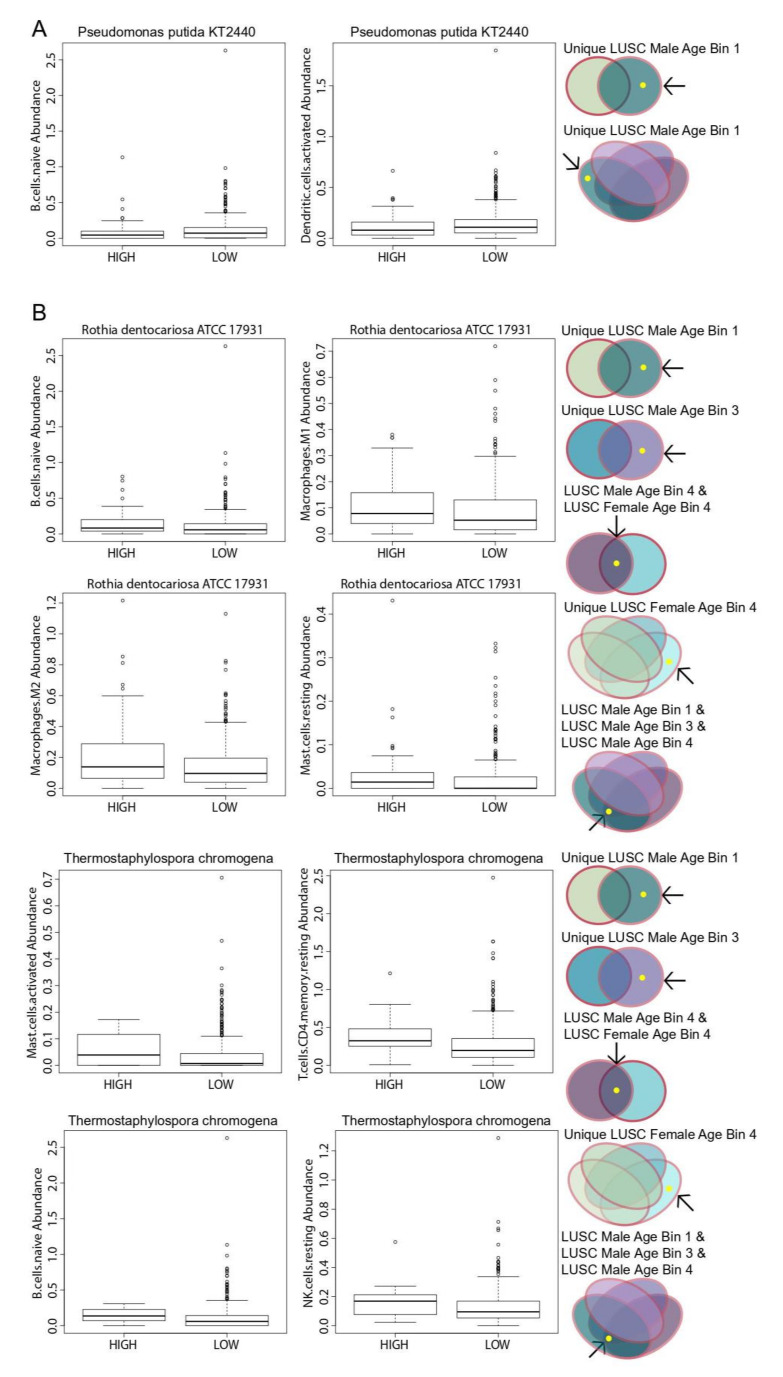
Correlation of differentially abundant microbes in LUSC to immune cell populations. Boxplots showing that a higher expression of microbes correlates to (**A**) a lower abundance of immune cell populations and a (**B**) higher abundance of immune cell populations. The comparisons and cohorts that these microbes are associated with are shown to the side.

We also correlated the abundance of the same set of significant microbes to immune cell infiltration using the Kruskal–Wallis test (*p* < 0.05). To quantify immune infiltration, we inferred immune cell levels using the CibersortX program, which deconvolves bulk RNA-sequencing data using gene expression signatures of immune cell activities. The relative levels of 22 immune cells in the tumor were inferred, and the immune cell abundances were correlated to microbe abundance using the Kruskal–Wallis test (*p* < 0.05). Only three LUSC-associated microbes were found to correlate with immune cells: higher abundance of one LUSC-associated microbe and lower abundance of two LUSC-associated microbes were associated with lower infiltration levels of certain immune cell populations. A higher abundance of *P. putida* str. KT2440 was correlated with the lower infiltration of both naive B cells and activated dendritic cell populations (Figure 5A). On the other hand, a lower abundance of *R. dentocariosa* str. ATCC 17931 was associated with lower infiltration levels of naive B cells, M1 macrophage, M2 macrophage and resting mast cell populations (Figure 5B). A lower abundance of *T. chromogena* was also associated with lower infiltrations levels of immune cell populations, including activated mast cells, resting CD4 T cells, naive B cells and resting natural killer (NK) cell populations. 

The microbe P. putida KT2440 is associated uniquely with LUSC Male Age Bin 1 for both the gender and age bin comparisons. For the gender comparisons, *R. dentocariosa* str. ATCC 17931 and *T. chromogena* are uniquely associated with LUSC Male Age Bin 1 and LUSC Male Age Bin 3 and in common with LUSC Male and Female Age Bin 4 (Figure 1D). For the age bin comparisons, *R. dentocariosa* str. ATCC 17931 and *T. chromogena* are uniquely associated with LUSC Female Age Bin 4 and in common between LUSC Male Age Bin 1, 3, and 4 (Figure 1E). 

### 2.8. Correlation of Abundance of Significant Microbes to Immune Activity Signatures

**Figure 6:**
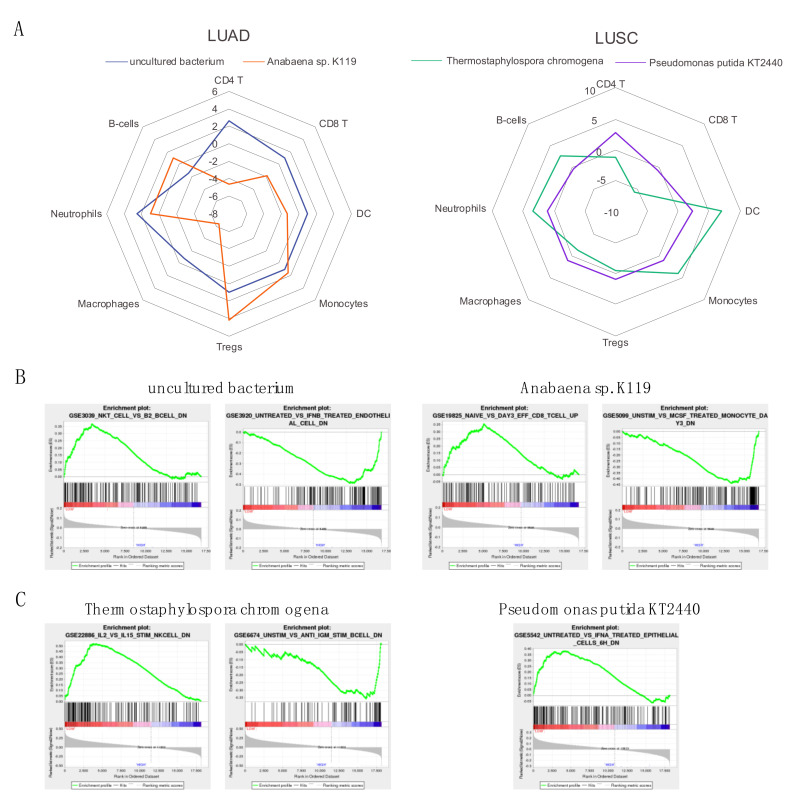
Association between differentially abundant microbes in LUAD and LUSC and immune signatures. Only the two most differentially abundant microbes in each cancer are included (Kruskal–Wallis test, *p* < 0.05). (**A**) Radar plot demonstrating the sum of the -log(*p*-value) or log(*p*-value) of immune and cancer pathways that have positive and negative correlations, respectively, with microbe abundance and are associated with the immune cell types depicted. (**B**) Select GSEA plots of immunologic signatures for the two most significant microbes in LUAD and (**C**) LUSC (Kruskal–Wallis test, *p* < 0.05). A peak indicates that a higher abundance of the microbe correlates with a higher expression of the genes in the gene set whereas a valley indicates that lower abundance of the microbe correlates to lower expression of the genes in the gene set.

We chose two of the most significant microbes per cancer according to their associated *p*-value from comparing all LUAD or LUSC patients to their corresponding normal patients. We first correlated the abundance of the chosen microbes to immune-associated signatures from the Molecular Signatures Database (MsigDB) using Gene Set Enrichment Analysis (GSEA) (*p* < 0.05). Using the immune-associated signatures, we were able to deduce the association between each microbe and the activity of CD4 T cells, B cells, CD8 T Cells, dendritic cells, neutrophils, macrophages, monocytes and regulatory T cells. For the immune signatures associated with one of the eight cell types mentioned earlier, we summed the -log (*p*-value) of positively associated immune signatures with the log (*p*-value) of negatively associated immune signatures for each microbe and visualized this metric in a radar plot to indicate the activity of each immune cell population (Figure 6A). The summed value of 1 is defined as the microbial abundance levels having no association with the immune cell type. The LUAD-associated microbe *uncultured bacterium* was found to be positively associated with CD4 T cells, neutrophils and regulatory T cells and negatively associated with B cells. The abundance levels of *Anabaena* sp. K119, which is also associated with LUAD, were discovered to be positively correlated with regulatory T cells and monocytes and negatively correlated with CD8 T cells, dendritic cells, macrophages and CD4 T cells. For LUSC, *T. chromogena* was found to be positively associated with B cells, neutrophils, monocytes and dendritic cells and negatively associated with CD8 T cells, regulatory T cells, macrophages and CD4 T cells. On the other hand, the LUSC-associated microbe *P. putida* str. KT2440 was associated with fewer microbes, including positively correlating with CD4 T cells and dendritic cells and negatively correlating CD8 T cells and B cells. 

For each of the four microbes, we attempted to select GSEA plots that were positively enriched based on high and low abundance of the selected microbes. For LUAD, a high abundance of *uncultured bacterium* was associated with higher activity of NKT cells compared to activity of B2 B-cells and higher exposure to IFNB (Figure 6B). High abundance of *Anabaena* sp. K119 was found to correlate with a higher activity of effector CD8 T cells compared to naïve T cells and higher activity of MCSF-activated monocytes compared to unstimulated monocytes. For LUSC, high abundance of *T. chromogena* was associated with higher activity of IL2-stimulated NK cells compared to IL15-stimulated NK cells and higher activity of anti-IGM stimulated B cells compared to unstimulated B cells (Figure 6C). Lastly, a high abundance of *P. putida* str. KT2440 correlates to a higher activity of untreated epithelial cells compared to epithelial cells exposed to interferon-alpha (IFNA). 

Both *uncultured bacterium* and *Anabaena* sp. K119 are in common between all LUAD gender comparisons and age bin comparisons. In LUSC gender comparisons, *T. chromogena* is found in common between LUSC Female Age Bin 4 and LUSC Male Age Bin 4 and unique to LUSC Male Age Bin 1 and LUSC Male Age Bin 3, whereas, in the LUSC age bin comparisons, it is found in common between LUSC Male Age Bin 1,3,4 and unique to LUSC Female Age Bin 4. As mentioned previously, *P. putida* str. KT2440 is unique to LUSC Male Age Bin 1 in both the LUSC gender and age bin comparisons. While we only presented four microbes here, we have included raw GSEA results for all significant age and gender-associated microbes as supplementary data (Supplementary Data 1). 

### 2.9. Association of Significant Microbial Abundance to Cancer and Immune Pathways

**Figure 7:**
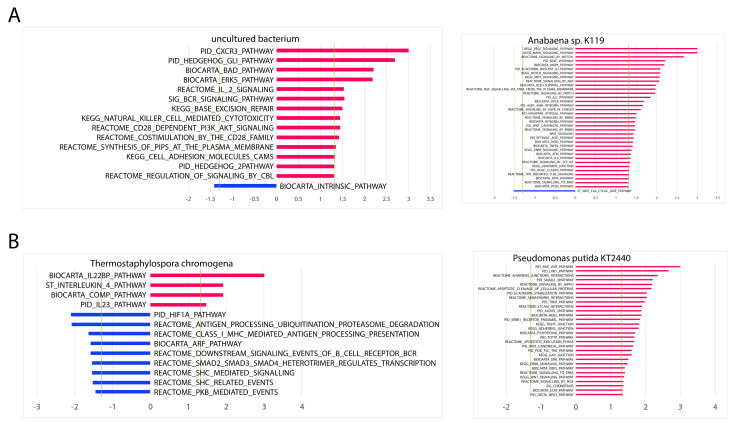
Association between differentially abundant microbes in LUAD and LUSC to cancer and immune pathways. Bar plot of the log(*p*-value) for positive associations and -log(*p*-value) for negative associations for significant cancer and immune pathways (GSEA, *p* < 0.05) in (**A**) LUAD and (**B**) LUSC. Green vertical lines indicate the significance cut-off of log(0.05) and −log(0.05), where values falling outside of the region bordered by the green lines indicate significance. Only the two most differentially abundant microbes in each cancer are included (Kruskal–Wallis test, *p* < 0.05).

Using the same GSEA analysis, we also correlated the abundance of these four microbes to significant cancer and immune pathways (*p* < 0.05) (Figure 7). Overall, *uncultured bacterium* and *T. chromogena* had the fewest number of associated cancer and immune pathways. Most of the microbes shown had a greater proportion of positively associated pathways. *P. putida* str. KT2440 is only positively associated with significant cancer and immune pathways. However, *T. chromogena* had a greater proportion of negatively associated pathways in comparison to the positively associated pathways.

In terms of proportions of cancer to immune pathways, LUAD-associated microbe *uncultured bacterium* and LUSC-associated microbe *T. chromogena* were correlated to approximately equal numbers of cancer and immune pathways. Both the LUAD-associated microbe *Anabaena* sp. K119 and the LUSC-associated microbe *P. putida* str. KT2440 were correlated to at least four times as many cancer pathways than immune pathways.

### 2.10. Correlation of Genomic Alterations to Significant Microbial Abundance

**Figure 8:**
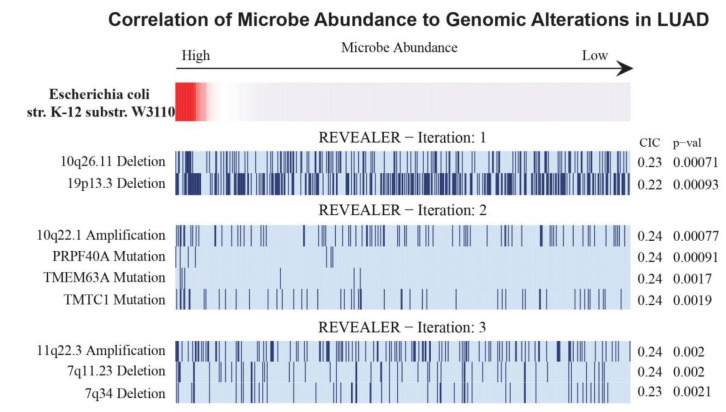
Association between genomic alterations and the abundance of a microbe in LUAD using Repeated Evaluation of Variables conditionAL Entropy and Redundancy (REVEALER) (CIC > 0.2, *p* < 0.05).

Out of the significant microbes following the gender and age bin comparisons for LUAD and LUSC, only one LUAD-associated microbe was found to be significantly associated with genomic alterations. A high microbial abundance of *E. coli* str. K-12 substr. W3110 is correlated with four deletion loci, three mutation loci and one amplification loci using the criteria of CIC > 0.2 and *p*-value < 0.05 (Figure 8). These genomic alterations include 10q26.11 (deletion), 19p13.3 (deletion), 7q11.23 (deletion), 7q34 (deletion), PRPF40A (mutation), TMEM63A (mutation), TMTC1 (mutation) and 10q22.1 (amplification). 

## 3. Discussion

Ongoing research and recent studies have indicated that the microbiome plays an important role in regulating and contributing to the progression of cancer [16]. Although most studies have focused primarily on the gut microbiome in relation to cancer, several studies have begun attempting to identify key microbial species that lead to cancer in other tissues, including the skin, colon, liver and lungs [17,18,19]. In particular, studies on the lung microbiome have correlated the lung microbiota in lung cancer with patient survival and established potential mechanisms for its relation to lung cancer progression via specific immune pathways [20,21]. A study by Greathouse et al. analyzed TCGA LUSC and LUAD data to discern the combinatorial effects of somatic mutations and smoking while adjusting for age and gender as co-variates, but the study did not identify microbes associated with age and gender [22]. In this study, we focus on identifying and comparing unique and common microbial species in a total of 16 LUAD and LUSC patient cohorts that are stratified according to age and gender using TCGA data and correlating the abundance of the associated microbes to patient survival, immune cell populations, immune signatures, immune and cancer pathways, and genomic alterations. To the best of our knowledge, we have not found any other study that comparatively analyzes the LUAD and LUSC-associated microbiota when taking age and gender into account.

After rigorously identifying and removing potential microbial contaminants, we separated LUAD and LUSC patients into eight cohorts each based on four different age groups and two genders and compared each cohort’s associated microbial abundance to the microbial abundance of corresponding adjacent normal samples. From this comparison, we took differentially abundant microbes in each cohort and performed gender comparisons and age bin comparisons, which involve contrasting genders with the same age bin and age bins within one gender, respectively, within individual cancers. We also compared between cancers for microbes uniquely associated with a certain cohort or in common with two or four cohorts. For all comparisons, LUSC cohorts generally had fewer differentially abundant microbes compared to LUAD cohorts. For gender comparisons, in particular, we noted that it was more common for LUAD samples to share differentially abundant microbes between genders in each age bin than LUSC samples. LUAD cohorts were more likely than LUSC cohorts to contain significant unique microbes with high abundance whereas only LUSC cohorts contained significant unique microbes with low abundance. We found that, for age bin comparisons, only LUAD contained a significant microbe with high and low abundance. LUAD also had a greater number of cohorts uniquely associated with microbes with high abundance than LUSC, which only had three cohorts. No unique microbes or microbes in common across all four age bins were shared between both cancers. 

Overall, when comparing between genders, all LUAD female age bins contained uniquely implicated microbes except for LUAD Female Age Bin 4 (73–88 years). All LUAD male age bins contained uniquely implicated microbes where most contained more uniquely associated microbes than their female counterparts except for LUAD Male Age Bin 2 (33–58 years). In the LUSC gender comparisons, only two LUSC female age bins and two LUSC male age bins contained uniquely implicated microbes, which were LUSC Female Age Bin 2 (62–67 years) and 4 (73–85 years) and LUSC Male Age Bin 1 (45–61 years) and 3 (68–72 years). The uniquely implicated microbes in most LUAD and LUSC cohorts could explain why certain genders are more prone to being diagnosed with either LUAD or LUSC.

When comparing between age bins, all LUAD female cohorts and LUAD male cohorts had at least one uniquely associated microbe except for LUAD Male Age Bin 4 (73–88 years). On the other hand, for LUSC age bin comparisons, only LUSC Female Age Bin 2 (*A. calcoaceticus* str. DSM 20,006 = CIP 81.8), LUSC Female Age Bin 4 (*P. putida* str. F1, *R. dentocariosa* str. ATCC 17931, *T. chromogena*) and LUSC Male Age Bin 1 contained uniquely implicated microbes (P. putida str. KT2440). 

The majority of cohorts in LUAD and LUSC, which contained unique significantly implicated microbes in gender and age bins comparisons, demonstrates that there are significant microbial differences between age groups and gender in both cancers. Thus, a panel of predictive biomarkers based on specific age groups and gender is needed to perform early diagnoses of lung cancers. In addition to the microbes we identified using bulk RNA sequencing, other microbial species could potentially be identified by using 16S sequencing as it can provide a more specific identification of bacteria species that were unable to be cultured. 

To assess the potential prognostic significance of age- and gender-associated microbes and generate hypotheses related to their function, we correlated the significant microbes that we compared in the gender and age bin comparisons to patient survival, immune cell populations, immune signatures, immune and cancer pathways and genomic alterations. In most analyses, only a select few microbes exhibited significant correlations where they belonged to either LUAD or LUSC. Based on the Cox proportional hazards regression model, we determined that the LUAD-associated microbe *S. aureus* displayed tumor-suppressive properties as it significantly increased patient survival rates when its abundance levels were high. On the other hand, *E. coli* str. K-12 substr. W3110, which is also an LUAD-associated microbe, may function like an oncogene in that patient survival rates were significantly decreased when its abundance levels were high. For correlations with immune cell populations, lower abundance levels of both *R. dentocariosa* str. ATCC 17931 and T*. chromogena* and higher abundance levels of *P. putida* str. KT2440, which are both LUSC-associated microbes, were correlated with lower expression of various immune cell populations. 

In contrast to the analyses that correlated microbial abundance to patient survival and immune cell populations, there were a large number of significant microbial associations with immune signatures and immune and cancer pathways. For the two most significant microbes for each cancer, which included *uncultured bacterium* and *Anabaena* sp. K119 for LUAD and *T. chromogena* and *P. putida* str. KT2440 for LUSC, we found that the abundance of these microbes is significantly associated with several immune signatures that are related to specific immune cell types. All four microbes were found to negatively associate with CD8 T cells and macrophages and positively associate with neutrophils and monocytes. Other immune cell types, including regulatory T cells and dendritic cells were positively correlated with at least one LUAD-associated microbe and negatively associated with at least one LUSC-associated microbe. We also selected examples of immune signatures that were significantly dysregulated as a result of low or high abundance of these four microbes. The abundance of the four microbes was also correlated to immune and cancer pathways. *Uncultured bacterium* and *T. chromogena* had the fewest associated cancer and immune pathways. Although three of the microbes had more positive associations than negative associations with immune and cancer pathways (*P. putida* str. KT2440 had all positive associations), *T. chromogena* exhibited the opposite proportions of positive to negative associations of immune and cancer pathways. Finally, we performed Repeated Evaluation of Variables conditionAL Entropy and Redundancy (REVEALER) to correlate the significant microbes following the gender and age bin comparisons to genomic alterations. Only *E. coli* str. K-12 substr. W3110, which is an LUAD-associated microbe, was significantly correlated with genomic alterations, which consisted of one amplification loci and mainly deletion and mutation loci. 

Out of the microbes that were significantly correlated to at least one analyses, only *E. coli* str. K-12 substr. W3110, *S. aureus*, *R. dentocariosa* str. ATCC 17931, and *P. putida* str. KT2440 were found in humans [23,24,25,26]. Because the majority of bacteria is unculturable, only a small fraction of the bacteria is represented in the data. For the microbe that appeared most frequently in our analyses, which was the LUAD-associated microbe *E. coli* str. K-12 substr. W3110, no other studies have found this microbe, or microbes closely related to it, to be implicated in the human microbiome and cancer. *E. coli* str. K-12 is commonly used as a model organism, and the substrain W3110 is used as a wild-type strain in experiments globally [26]. On the other hand, the LUAD-associated microbe *S. aureus*, which is a known pathogen that colonizes nasal areas, has been found to increase the risk of patients dying from cancer [25,27]. It has been discovered that the colonization of *S. aureus* occurs more frequently in men than women [28]. Furthermore, LUSC-associated microbes *R. dentocariosa* str. ATCC 17931, which is normally found in the oral cavity, was discovered to cause lung infections and pneumonia [24]. Finally, *P. putida* str. KT2440 was isolated from blood samples in cancer patients, including one lung cancer patient [23]. In conclusion, we have discovered novel associations for several bacteria known to be present in humans but never before correlated to gender and age in lung cancer. Our results could be critical to efforts to diagnose, treat, or prevent lung cancer using the microbiome composition. Lastly, gender and age difference in microbe levels may also contribute to biological differences in the mechanism of lung cancer. 

## 4. Materials and Methods

### 4.1. Acquisition of TCGA RNA-Sequencing Datasets

RNA-sequencing tumor tissue data for 497 LUAD and 433 LUSC patients along with adjacent solid tissue normal data for 49 LUSC patients and 59 LUAD patients was obtained from The Cancer Genome Atlas (https://portal.gdc.cancer.gov/legacy-archive/search/f) on 5 Aug 2018. Clinical information for each patient was downloaded from the Broad GDAC Firehose (https://gdac.broadinstitute.org/). Data related to genomic alterations for each patient were downloaded from the Broad Institute TCGA Genome Data Analysis Center’s (http://gdac.broadinstitute.org/runs/analyses__latest/reports/) analysis report (2016). Pathoscope 2.0 [14] was used to filter RNA-sequencing data for bacterial reads using direct alignment through Bowtie2. The NCBI’s nucleotide database was accessed for bacterial sequences. Pathoscope’s best hit output data, the absolute count of each species in the data, was used to measure the amount of bacterial species present in a sample.

### 4.2. Differential Microbial Abundance between Cancer and Normal Samples

Differential abundance analysis was performed to compare microbe abundance (percent abundance) in cancer tissues to microbe abundance in normal tissues of the same body site. Microbes that are present in less than 10 patients of the same cancer were excluded. The Kruskal–Wallis analysis test was then applied to determine differential abundance (*p* < 0.05).

### 4.3. Differential Microbe Abundance Based on Patient Age and Gender

Kruskal–Wallis testing was performed on microbial abundance for male cancer patients and female cancer patients in order to determine the association between abundance and gender. Patients were also divided into four age bins. The boundaries of the age bins were defined by the minimum age, maximum age, or one of the three quartiles when including ages of all patients of the same cancer. Kruskal–Wallis testing was performed on the microbe abundances vs. age bins to determine the association between abundance and age. Kruskal–Wallis tests were also performed on microbe abundances between 8 cancer patient groups, divided by age and gender. These included four age bins of female cancer patients and four age bins of male cancer patients. For both female and male patients in LUAD, age bins 1, 2, 3, and 4 correspond to age ranges of 33–58, 59–65, 66–72, 73–88, respectively. For both female and male patients in LUSC, age bins 1, 2, 3, and 4 correspond to age ranges of 45–61, 62–67, 68–72, 73–85, respectively. The number of patients in each patient group is listed in Table S3. The distribution of tumor stages for each age bin is listed in Table S4. For all groups, comparisons were between microbe abundances of the group and microbe abundances in normal tissue.

Associations found to be significant (*p* < 0.05) were separated based on whether microbe abundance was higher in a particular patient group or in normal tissue. Venn diagrams were made to show unique microbes and microbes in common between different combinations of groups. Diagrams included microbes that were differentially abundant in the same direction for both groups. Venn diagram comparisons were made between male patients and female patients in the same age bin with the same cancer, between patients in different age bins with the same gender and the same cancer, and between patients with the same age bin, the same gender and different cancers.

### 4.4. Visualization of Microbial Population Distribution Using PCoA 

We first determined that principle component analysis (PCA) and correspondence analysis (CA) were not ideal because of undesirable gradient and plot shape, respectively. The Bray–Curtis dissimilarity measure was then calculated for all cancer samples for LUAD and LUSC, and then PCoA was performed using the dissimilarity matrix as the input. Analyses were performed using R and the R package vegan. 

### 4.5. Identification of Smoking Associated Microbes in LUSC and LUAD

We identified smoking associated microbes by comparing the microbe abundance in cancer samples from smokers at time of diagnosis vs. that in cancer samples from life-long nonsmokers (χ^2^ test, *p* < 0.05). 

### 4.6. Logistic Regression to Correlate Clinical Variables with Microbe Abundance

To identify confounding variables, we correlated age and gender-associated microbes to other clinical variables using logistic regression. The variables included were ethnicity, pathologic stage, vital status, number of pack-years smoked, pathologic TNM stages, tobacco smoking history, and race. 

### 4.7. Correlation of Microbial Abundance to Patient Survival

Survival analyses were performed using the Kaplan–Meier model, with microbe expression designated as a binary variable based on the presence or absence of a microbe in tumor samples. Univariate Cox regression analysis was used to identify candidates that were significantly associated with patient survival (*p* < 0.05). 

### 4.8. Correlation of Microbial Abundance to Immune Infiltration

Estimated relative immune cell infiltration levels for 22 cell types were computed using the software Cibersortx [29]. Microbe abundance was then correlated with immune cell infiltration levels for each microbe using the Kruskal–Wallis test (*p* < 0.05). Microbe abundance was modeled as a binary variable of presence and absence. The immune cell types examined include naïve B-cells, memory B-cells, plasma cells, CD8 T-cells, CD4 naïve T-cells, CD4 memory resting T-cells, CD4 memory activated T-cells, follicular helper T-cells, regulatory T-cells, gamma-delta T-cells, resting NK cells, activated NK cells, monocytes, M0-M2 macrophages, resting dendritic cells, activated dendritic cells, resting mast cells, activated mast cells, eosinophils, and neutrophils.

### 4.9. Immune Pathway Association with Microbial Expression Using GSEA

GSEA was utilized to identify microbes associated with the dysregulation of biological pathways and signatures, which are obtained from the Molecular Signature Database (MSigDB) [30]. Specifically, canonical pathways (C2) and immunologic signatures (C7) were examined. The abundance data for each microbe were inputted as a categorical variable (presence or absence) in the phenotype file. The gene expression dataset consisted of the expression values of all genes in counts per million (CPM). Using Pearson’s correlation for the continuous phenotypes and signal-to-noise ratio for categorical phenotypes, the microbe abundance is correlated to the above gene sets to generate enrichment scores. Higher enrichment scores indicate stronger correlation between microbe abundance and expression of genes within a gene set.

### 4.10. REVEALER Association of Microbe Abundance with Genomic Alterations

The Repeated Evaluation of Variables conditionAL Entropy and Redundancy (REVEALER) program was used to identify a statistically significant association of genomic alterations (amplifications, deletions, or mutations) with the abundance of individual microbes. We define an association as significant if the absolute value of its Conditional Information Coefficient (CIC) value was greater than 0.2 and if the *p*-value was less than 0.05.

### 4.11. Evaluation of Possible Contamination Using Plates and Date of Sequencing

The abundance values of microbes were associated with the plates on which the samples were stored prior to sequencing using the Kruskal–Wallis test and visual examination of abundance differences between different plates using a boxplot. For visual examination, the microbe abundance for each sample was plotted in order of date sequenced on a dot plot. If the microbe is a contaminant, samples sequenced near the same date would have a similar overexpression of specific bacterial species. Therefore, we applied a heuristic algorithm based on divisive clustering using the DIANA R package to extract the sample ranges where this overexpression occurs, which allowed us to determine potential contaminants’ relationship with the sequencing date. 

## 5. Conclusions

By performing large-scale bioinformatics analyses, we aim to elucidate the effects of influential factors, such as age and gender, on the relationship between the lung microbiome and lung cancer. Using cohorts comprised of cancer patients that were stratified according to age and gender for both cancers, we identified differentially abundant microbes for each cohort after comparing them against normal samples. In both age bin and gender comparisons, we discovered that most cohorts contained uniquely associated microbes. After performing several computational analyses, we also found that a few differentially abundant microbes are correlated to patient survival rates, immune infiltration, immune and cancer pathways, and genomic alterations. Collectively, our results demonstrate the importance of accounting for age and gender when identifying clinically relevant microbes in the lung microbiome that may be implicated in lung cancer pathogenesis. 

## Supplementary Materials

The following are available online at https://www.mdpi.com/2072-6694/12/6/1447/s1, Figure S1: Heat Maps of Differentially Abundant Microbes in LUAD and LUSC, Table S1: Comparison of Microbes across Cancer Cohorts. Table S2: Multivariate analysis of microbe abundance vs. clinical variables. Table S3: Patient cohort size. Table S4: Pathologic Stage of LUAD and LUSC Cancer Cohorts.

## References

[1] Youlden D.R., Cramb S.M., Baade P.D. (2008). The International Epidemiology of Lung Cancer: Geographical distribution and secular trends. J. Thorac. Oncol..

[2] Molina J.R., Yang P., Cassivi S.D., Schild S.E., Adjei A.A. (2008). Non-small cell lung cancer: Epidemiology, risk factors, treatment, and survivorship. Mayo Clin. Proc..

[3] Zhang N., Wang H., Xie Q., Cao H., Wu F., Di Wu D.B., Wan Y. (2019). Identification of potential diagnostic and therapeutic target genes for lung squamous cell carcinoma. Oncol. Lett..

[4] Li Y., Ge D., Gu J., Xu F., Zhu Q., Lu C. (2019). A large cohort study identifying a novel prognosis prediction model for lung adenocarcinoma through machine learning strategies. BMC Cancer.

[5] Erb-Downward J.R., Thompson D.L., Han M.K., Freeman C.M., McCloskey L., Schmidt L.A., Young V.B., Toews G.B., Curtis J.L., Sundaram B. (2011). Analysis of the lung microbiome in the “healthy” smoker and in COPD. PLoS ONE.

[6] O’Dwyer D.N., Dickson R.P., Moore B.B. (2016). The Lung Microbiome, Immunity, and the Pathogenesis of Chronic Lung Disease. J. Immunol..

[7] Maddi A., Sabharwal A., Violante T., Manuballa S., Genco R., Patnaik S., Yendamuri S. (2019). The microbiome and lung cancer. J. Thorac. Dis..

[8] Coburn B., Wang P.W., Diaz Caballero J., Clark S.T., Brahma V., Donaldson S., Zhang Y., Surendra A., Gong Y., Elizabeth Tullis D. (2015). Lung microbiota across age and disease stage in cystic fibrosis. Sci. Rep..

[9] Yatsunenko T., Rey F.E., Manary M.J., Trehan I., Dominguez-Bello M.G., Contreras M., Magris M., Hidalgo G., Baldassano R.N., Anokhin A.P. (2012). Human gut microbiome viewed across age and geography. Nature.

[10] Taneja V., Legato M.J. (2017). Microbiome: Impact of Gender on Function & Characteristics of Gut Microbiome.

[11] Xavier J.B., Young V.B., Skufca J., Ginty F., Testerman T., Pearson A.T., Macklin P., Mitchell A., Shmulevich I., Xie L. (2020). The Cancer Microbiome: Distinguishing Direct and Indirect Effects Requires a Systemic View. Trends Cancer.

[12] De Groot P.M., Wu C.C., Carter B.W., Munden R.F. (2018). The epidemiology of lung cancer. Transl. Lung Cancer Res..

[13] Wakelee H.A., Chang E.T., Gomez S.L., Keegan T.H., Feskanich D., Clarke C.A., Holmberg L., Yong L.C., Kolonel L.N., Gould M.K. (2007). Lung cancer incidence in never smokers. J. Clin. Oncol..

[14] Hong C., Manimaran S., Shen Y., Perez-Rogers J.F., Byrd A.L., Castro-Nallar E., Crandall K.A., Johnson W.E. (2014). PathoScope 2.0: A complete computational framework for strain identification in environmental or clinical sequencing samples. Microbiome.

[15] Morton J.T., Toran L., Edlund A., Metcalf J.L., Lauber C., Knight R. (2018). Correction for Morton et al., “Uncovering the Horseshoe Effect in Microbial Analyses”. MSystems.

[16] Whisner C.M., Athena Aktipis C. (2019). The Role of the Microbiome in Cancer Initiation and Progression: How Microbes and Cancer Cells Utilize Excess Energy and Promote One Another’s Growth. Curr. Nutr. Rep..

[17] Fessler J., Matson V., Gajewski T.F. (2019). Exploring the emerging role of the microbiome in cancer immunotherapy. J. Immunother. Cancer.

[18] Gopalakrishnan V., Helmink B.A., Spencer C.N., Reuben A., Wargo J.A. (2018). The Influence of the Gut Microbiome on Cancer, Immunity, and Cancer Immunotherapy. Cancer Cell.

[19] Schwabe R.F., Jobin C. (2013). The microbiome and cancer. Nat. Rev. Cancer.

[20] Gomes S., Cavadas B., Ferreira J.C., Marques P.I., Monteiro C., Sucena M., Sousa C., Vaz Rodrigues L., Teixeira G., Pinto P. (2019). Profiling of lung microbiota discloses differences in adenocarcinoma and squamous cell carcinoma. Sci. Rep..

[21] Jin C., Lagoudas G.K., Zhao C., Bullman S., Bhutkar A., Hu B., Ameh S., Sandel D., Liang X.S., Mazzilli S. (2019). Commensal Microbiota Promote Lung Cancer Development via gammadelta T Cells. Cell.

[22] Greathouse K.L., White J.R., Vargas A.J., Bliskovsky V.V., Beck J.A., von Muhlinen N., Polley E.C., Bowman E.D., Khan M.A., Robles A.I. (2018). Interaction between the microbiome and TP53 in human lung cancer. Genome Biol..

[23] Anaissie E., Fainstein V., Miller P., Kassamali H., Pitlik S., Bodey G.P., Rolston K. (1987). Pseudomonas putida. Newly recognized pathogen in patients with cancer. Am. J. Med..

[24] Khan S.T., Ahamed M., Musarrat J., Al-Khedhairy A.A. (2014). Anti-biofilm and antibacterial activities of zinc oxide nanoparticles against the oral opportunistic pathogens Rothia dentocariosa and Rothia mucilaginosa. Eur. J. Oral. Sci..

[25] Laux C., Peschel A., Krismer B. (2019). *Staphylococcus aureus* Colonization of the Human Nose and Interaction with Other Microbiome Members. Microbiol. Spectr..

[26] Riley M., Abe T., Arnaud M.B., Berlyn M.K., Blattner F.R., Chaudhuri R.R., Glasner J.D., Horiuchi T., Keseler I.M., Kosuge T. (2006). Escherichia coli K-12: A cooperatively developed annotation snapshot--2005. Nucleic Acids Res..

[27] Gotland N., Uhre M.L., Mejer N., Skov R., Petersen A., Larsen A.R., Benfield T., Danish Staphylococcal Bacteremia Study Group (2016). Long-term mortality and causes of death associated with Staphylococcus aureus bacteremia. A matched cohort study. J. Infect..

[28] Sangvik M., Olsen R.S., Olsen K., Simonsen G.S., Furberg A.S., Sollid J.U. (2011). Age- and gender-associated Staphylococcus aureus spa types found among nasal carriers in a general population: The Tromso Staph and Skin Study. J. Clin. Microbiol..

[29] Chen B., Khodadoust M.S., Liu C.L., Newman A.M., Alizadeh A.A. (2018). Profiling Tumor Infiltrating Immune Cells with CIBERSORT. Methods Mol. Biol..

[30] Liberzon A., Subramanian A., Pinchback R., Thorvaldsdottir H., Tamayo P., Mesirov J.P. (2011). Molecular signatures database (MSigDB) 3.0. Bioinformatics.

