# Supplementary material for: Comparative Analysis of Age- and Gender-Associated Microbiome in Lung Adenocarcinoma and Lung Squamous Cell Carcinoma
Source: Cancers (Basel). 2020 Jun 2;12(6):1447. doi: 10.3390/cancers12061447 (PMC7352186; doi:10.3390/cancers12061447)
Supplement: Supplementary file 1 [file cancers-12-01447-s001.zip › cancers-813686-suppl-proofreading/cancers-813686-suppl-proofreading.docx]

Article

Comparative Analysis of Age- and Gender-Associated Microbiome in Lung Adenocarcinoma and Lung Squamous Cell Carcinoma

Lindsay M. Wong, Neil Shende, Wei Tse Li, Grant Castaneda, Lauren Apostol, Eric Y. Chang and Weg M. Ongkeko

**Figure S1.** Heat Maps of Differentially Abundant Microbes in LUAD and LUSC.

| 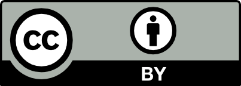 | © 2020 by the authors. Submitted for possible open access publication under the terms and conditions of the Creative Commons Attribution (CC BY) license (http://creativecommons.org/licenses/by/4.0/). |
| --- | --- |
